# Measuring the effect of employment uncertainty on fertility in low-fertility contexts: an overview of existing measures

**DOI:** 10.1186/s41118-023-00185-x

**Published:** 2023-02-07

**Authors:** Brian Buh

**Affiliations:** Austrian Academy of Sciences, Vienna Institute of Demography, Dr. Ignaz-Seipel-Platz 2, 1010 Vienna, Austria

**Keywords:** Employment uncertainty, Fertility, Job instability, Measuring uncertainty

## Abstract

Numerous studies aim to connect negative fertility desires and outcomes with employment conditions deemed to be uncertain. However, there is a lack of consensus about how to define, conceptualise, and measure employment uncertainty. This paper considers issues surrounding the conceptualisation of employment uncertainty. It then reviews existing measures of employment uncertainty in the context of fertility decisions. Finally, it raises considerations about their use. While some aspects of employment uncertainty are well studied, there are still gaps between theory and empirical evidence. Researchers should be aware of existing population heterogeneity, contextual factors, and model selection when considering their conceptualisation of employment uncertainty.

## Introduction

Growing employment uncertainty in advanced economies and its relationship to fertility has received significant attention in recent decades (Alderotti et al., 2021; Kreyenfeld et al., 2012; Matysiak & Vignoli, 2008). However, diverging results and complex interdependencies compel the need to review the full scope of approaches and measurements. Previous fertility research approaches employment uncertainty as a micro-level objective measures of employment, subjective perceptions, structural components, life-long trends, and emerging trends within countries. The lack of consistent definition and conceptualisation of employment uncertainty has led authors to make broad claims about the impact of employment uncertainty on fertility while their research may only cover one or two aspects.

Evidence from the 2008 recession shows that fertility rates dropped more in regions with higher unemployment rates and where labour market conditions deteriorated at a higher rate than in regions less affected by the recession (Matysiak et al., 2020). Yet, even countries that emerged from the recession largely unscathed have seen a decline in total fertility rates (TFR). For example, Norway saw fertility rates drop from 1.98 in 2009 to 1.6 in 2018 (Hellstrand et al., 2021). Additionally, TFRs have not recovered in Southern and Eastern European countries with lowest-low fertility rates (TFR 1.3 or lower) despite their economic recoveries (Comolli, 2017). Economic trends and unemployment are interlinked but not synonymous and may not explain micro-level reactions to changes in economic conditions. Studies using macro-indicators help clarify observed changes in TFR and are particularly fit for cross-country comparison. However, they have difficulty examining the mechanisms of changes in fertility behaviour.

A growing volume of literature examines micro-level employment conditions to explain the mechanisms behind the continued decrease in fertility. Early theories addressing the role of employment on fertility and childbearing focus on the effects of unemployment. The microeconomic model of fertility theorises that unemployment produces two competing effects on the demand for children (Becker, 1960). On the one hand, the *income effect* constrains the demand for children by reducing available financial resources needed for childrearing. On the other hand, the *substitution effect* lowers the opportunity cost of childbearing, thus increasing the demand for children. Both effects have been observed empirically, although the substitution effect has been primarily observed for women (Adsera, 2004). Evidence from a meta-analysis of studies looking at the effect of unemployment on fertility rates indicates that the effect unemployment has on reducing fertility outcomes in Europe has become stronger over the last few decades (Alderotti et al., 2021). Additionally, the substitution effect appears to be weakening, with employment being a more important precursor than unemployment for women’s fertility desires.

Employment uncertainty is, however, much broader than just unemployment. Several approaches have emerged to measure employment uncertainty within the realm of fertility research. Theory in the fields of uncertainty and fertility suggests that interlinkages of life course domains, past experiences, and future perceptions make simple indicators insufficient (Bernardi et al., 2019; Vignoli et al., 2020a). Recent attempts have been made to create indicators that study the effects of uncertainty based on individual employment histories and perceptions of future uncertainty (Busetta et al., 2019; Fahlén & Oláh, 2018). Considering the complications around conceptualising and measuring employment uncertainty, this paper aims to provide a broad overview of previous employment uncertainty measures in the field of fertility in Europe. It assesses these issues by conceptualising employment uncertainty, reviews the indicators introduced in the literature, briefly looks at broad conclusions based on evidence from these indicators, and discusses modelling considerations.

The paper is structured as follows: "Concepts" section looks at the conceptualisation of employment uncertainty and what effect this has on methodology. "Measures" section describes the main indicators introduced in the literature. "Discussion" section discusses considerations when modelling the link between fertility and employment uncertainty, and "Conclusion" section concludes.

## Concepts

The definition of uncertainty remains debated within fertility research. Vignoli et al. (2020a) classify definitions of general life course uncertainty into three categories: *social interaction*, *available information*, and *fundamental uncertainty*. Uncertainty by *social interaction* is defined as the inability to predict the behaviour of others since all individuals are simultaneously learning and adapting (Elster, 2009). Uncertainty results from a lack of *available information* when individuals have neither the ability nor the time to collect information nor are unaware of its existence (Davidson, 1996). In both cases, the uncertainty arises from the inability to properly forecast future risks or probabilities.

*Fundamental uncertainty* is defined as uncertainty which arises from future outcomes that inherently cannot be forecasted or even potentially known (Beckert, 2016). Concerning employment, this type of uncertainty, studied through the lens of globalisation, deregulation, and technology, started to gain prominence in the 1980s (Hacker, 2019). Substantial transformations in labour market dynamics since then have profoundly changed structural and individual employment security (Gebel & Giesecke, 2011; Mills & Blossfeld, 2003; Peters, 2008). For example, market internationalisation makes them increasingly relevant to all aspects of life, making employment more sensitive to the dynamic nature of price and competition (Beck, 1992; Gottfried, 2014). However, fundamental uncertainty may affect individuals’ behaviour differently depending on their capacity and willingness to accept the unknown. Most individuals will experience uncertainty at some point, but when, how, and the scale in which they experience it varies greatly (Mayer, 2009). Intensified competition additionally contributes to uncertainty by increasing the need to acquire more human capital, usually through increased education, postponing entry into parenthood (Blossfeld et al., 2006; Kohler et al., 2002). When linking employment uncertainty and fertility, it is this postponement effect as well as the proximity of uncertainty surrounding childbearing decisions that affect fertility intention (Bernardi et al., 2015). With the multiple manifestations of uncertainty in mind, researchers need to clearly define what they specifically want to study.

Using the above classifications of uncertainty, we define employment uncertainty as: *Individuals experience employment uncertainty when employment stability is not, or is not perceived as, guaranteed, or when current employment is viewed as inadequate to achieve other life course goals* (Anderson & Pontusson, 2007; Esser & Olsen, 2012). Employment uncertainty is by definition subjective. However, employment uncertainty is, at large, measured objectively (fixed-term contracts, seasonal or temporary employment, volatile self-employment, or involuntary part-time employment). Even individuals with stable employment conditions can still feel that their job has inadequate remuneration, advancement opportunities, flexibility, scheduling, benefits, or prestige. Micro-level suboptimal employment may create the perception of employment uncertainty. There is significant occupational psychology literature on *job instability*, which is a similar concept with a similar definition (Cheng & Chan, 2008; Sverke et al., 2002). However, it largely focuses on employed individuals’ perception of the likelihood to lose current employment.

However, the above definition does not provide a clear guideline on how to measure employment uncertainty. Most measurements fall into two larger categories: *objective measures*, which look at previous or current employment situations and *subjective measures*, which ask individuals to evaluate perceived present and future risks. These measures can be further subcategorised as *macro* and *micro* measures.

*Objective measures* of employment uncertainty focus on individuals’ risk factors like employment status, contract type, or job characteristics. Many of the measures have similar definitions adopted by large data-collecting agencies and are available from a variety of data sources (e.g., registries, censuses, or surveys). National governments and/or private entities within countries (e.g., insurance companies) often collect vital statistics and employment status on a yearly, monthly, or even weekly basis, making large, reliable datasets available. Harmonised definitions allow objective measures to have advantages for cross-country comparisons. Hence, it is the universality, availability, comparability, and perceived straightforwardness that makes them attractive. However, objective measures are not inherently more straightforward, especially when used to measure subjective concepts like employment uncertainty. Almost all other objective measures of employment uncertainty build upon how researchers demarcate employment statuses as either certain or uncertain. Therefore, a study’s conceptualisation of employment status matters. While objective measures may be preferred because they lack the pitfalls of interpersonal subjectivity, they also do not capture differences in the experience or perception of employment uncertainty—something better fit for subjective measures.

*Subjective measures* are generally elicited through individual surveys, which include questions about levels of perceived employment security or proxied via associated stressors. This includes financial and employment security, job-related stressors, and/or job loss resiliency (Begall & Mills, 2011; Fahlén, 2013; Fahlén & Oláh, 2018). Subjective measures more directly capture the feeling of uncertainty. However, these measures have several disadvantages. For example, subjective measures are only as applicable as the survey question’s proper use. Survey questions are often asked with a specific model in mind (e.g., Theory of Planned Behaviour) (Brehm & Schneider, 2019). The questions’ wording may not fit into the researcher’s desired conceptualisation of employment uncertainty. They are also sensitive to recent events, respondents’ immediate circumstances, and cultural and linguistic bias (Jahedi & Méndez, 2014). Even in the same cultural context, interpersonal differences like optimism or pessimism complicate comparing perceived levels of uncertainty. While the obvious solutions might seem to be using both objective and subjective measures in the same model, doing so often overestimates the effect of the explanatory variables. Researchers must carefully examine the proposed mechanism linking different measures of employment uncertainty and fertility before interpreting results.

From this understanding, it is clear that employment uncertainty arises from individual (micro) experiences. They can, however, also be influenced by community or society-wide changes. Therefore, *macro measures* may be applicable for studying employment uncertainty. Macro-measures are often an aggregate of individual-specific measurements, but some influential measures are computed directly at the macro-level (e.g., consumer confidence index). This includes measures like national and local unemployment rates as well as the share of specific contractual agreements (e.g., share of public sector employees, part-time work, or fixed-term contracts) (Adsera, 2004). Recent work has started to look at how macro-subjective perceptions of employment uncertainty (at the community and national level) influence individual fertility behaviour (Vignoli et al., 2020a).

Finally, employment uncertainty differs from concepts like *income volatility* and *economic insecurity*. Income volatility is defined as income fluctuations from a trend, generally measured as deviations from a predicted mean, and is a backwards-looking objective measure (Iceland, 2005). While income volatility might overlap with periods of employment uncertainty, the two concepts differ (e.g., a self-employed person may have a highly volatile income, but may not feel insecure about their employment). Moreover, economic insecurity, oftentimes conflated with employment uncertainty, is a much broader topic that incorporates the individual’s financial and human capital and their ability to handle economically difficult times and escape financially tenuous situations (Richiardi & He, 2019). For example, highly educated persons may experience long periods of employment uncertainty, particularly early in their careers, but not suffer from significant economic insecurity, while the opposite might be true for the less educated (Stiglitz et al., 2009). Thus, it is important to note the difference between employment uncertainty, economic insecurity, and income volatility.

## Measures

Considering the complexity involved in defining and conceptualising employment uncertainty, as outlined above, this section reviews employment uncertainty measures identified in the fertility literature. It starts with micro-objective measures ("Employment status"–"Occupational class" sections), proceeding to micro-subjective measures ("Perceived job characteristics"–"Experimental methods" sections), and concluding with macro measures ("Macro-measures" sections). Measures are summarised in Table 1 in Appendix.

We identified articles by searching academic databases (Scopus and Google Scholar). We screened the articles using screening and eligibility criteria.^1^ Several articles use similar measures. In such cases, we select the article in which the measure first appeared. As such, we aim to provide a full scope of existing measures rather than an exhaustive list of articles researching the subject.

### Employment status

Numerous studies analyse individuals’ or couples’ employment status in the context of fertility/childbearing. Baizán (2005) explores the effect of employment status nine months before childbirth and differentiates between employed, unemployed, student, or housewife. Additionally, he includes the employment sector (public or private), contract type (stable, temporary, or self-employed), and working hours (full or part-time) collected once per year and assumed to apply consistently over that year. Baizán (2009) further revises this model and includes a time-varying employment status, which is updated monthly. Adsera (2011b) examines women’s employment status over the previous seven months and distinguishes between working and unemployed/inactive. Work is further differentiated by creating dummy variables for part-time work, public sector, self-employed, and very short contracts.

Barbieri et al. (2015) create four categories of workers using employment types, consisting of *permanent employment*, *self-employment*,^2^*atypical employment*, and other *non-standard job*s.^3^ They construct this typology using the type of contract for dependent workers (permanent, fixed-term, training contract, seasonal, or off-the-books), manner of self-employment (with employees, without employees, freelancer, or entrepreneur), whether the work was seasonal or occasional, and the standardised four-digit International Standard Classification of Occupation (ISCO) code.^4^ To capture employment uncertainty, the authors create a binary indicator for the transition from an insecure to a secure employment position using the four established employment types, where *0* indicates insecure work (atypical or non-standard) and *1* indicates secure work (permanent or self-employed).

An alternative approach categorises the positional status of a woman in the household by accounting for her partnership status and her partner’s employment status. Alongside women’s employment status, Wood and Neels (2017) create 11 time-constant^5^ categories: (1) child in the household, (2) single, (3) married to an employed partner, (4) married to an unemployed partner, (5) married to an inactive partner, (6) married to a partner of unknown status, (7) cohabitating with an employed partner, (8) cohabitating with an unemployed partner, (9) cohabitating with an inactive partner, (10) cohabitating with a partner of unknown status, and (11) other. Comolli (2021) builds a categorical variable with couple-level employment and relative income situation. This includes: (1) dual-earner both full-time, (2) dual-earner one part-time, (3) male breadwinner, (4) female breadwinner, (5) man single-earner and woman unemployed, (6) woman single earner and man unemployed, and (7) dual jobless.

The above-mentioned studies focus on current employment. Vignoli et al. (2020c) examine the effect of early-career uncertainty by separating the first job into permanent versus temporary, fixed-term, or project-based. They use a matching system to compare men and women holding different types of employment positions in the first 5 years of their careers to determine if specific contract situations relate to the likelihood of becoming a parent.

### Number and duration of job spells

Using retrospective employment histories is an approach to quantify time spent in unemployment (e.g., total number of months spent in unemployment, ratio of years/months spent in paid employment, number of previous job shifts). Özcan et al. (2010) analyse birth outcomes by studying the number of months spent in unemployment, the number of unemployment spells, and the number of prior job shifts. Schmitt (2008) takes a similar approach by adding the number of incidences of long-term employment (spells longer than four months) within the last five years. Schmitt (2021) uses a time-varying indicator of the percentage of months since age 25 unemployed. The indicator takes the total number of months unemployed over the number of months since the respondents’ 25th birthday.

Pailhé and Solaz (2012) examine employment uncertainty by creating three indicators to explore both timing of births and completed fertility. The first is a time-varying indicator that looks at employment status in the previous year: long-term employment, short-term employment, unemployment, or homemaker (for women). It is measured at *t-1* to capture the recent experiences of each individual in the labour market. Next, they create a time-varying ratio of years spent in unemployment/short-term employment over permanent employment since union formation. Finally, they include employment status at union formation.

In contrast to measuring unemployment as a count of states or as a ratio, other work attempts to measure more dynamically by combining the number and intensities of unemployment spells into one indicator. Ciganda (2015) uses the notion of a stable state^6^ as a desired state and then uses sequence analysis to generate a unique indicator of time spent in unstable states,^7^ combining previous count and ratio measures.

Busetta et al. (2019) use a similar approach for their *Persistent Joblessness Indicator* (PJI), which standardises the experience of career joblessness between *0* and *1*. Their indicator focuses on being in or out of paid employment as a binary state. Like Ciganda (2015), they use sequence analysis to combine the number and intensity of joblessness spells. However, the PJI contributes to Ciganda’s method by additionally calculating the proximity of years with jobless periods as well as the recentness of joblessness to the observation period. Finally, they factor in local labour market characteristics by controlling for the unemployment experiences of peers.

### Occupational class

The relationship between employment uncertainty and fertility is likely mediated by occupational class. Bernardi and Nazio (2005) apply the International Standard Classification of Occupations, stratifying them into eight groups: *service*, *routine white collar*, *skilled worker*, *unskilled worker*, *unskilled manual worker*, *self-employed with employees*, *self-employed without employees*, and *agricultural worker.* The authors consider individuals who are unskilled workers, self-employed, or agricultural workers as being more economically insecure since they generally have lower incomes and are less able to save. They link this to employment uncertainty by also controlling for contract type (permanent contract, fixed-term training contract, other forms of fixed-term contract, consulting job, or without a contract) as well as employment status (in education, out of education but not looking for a first job, in search of the first job, unemployed, inactive, and employed).

### Perceived job characteristics

Perceptions of job flexibility, control, and tasks help clarify the relationship between participation in the labour market, hours worked, and the perception of employment uncertainty. Recent work examines job characteristics like working location, perceived work–life balance, and job-related well-being (Kurowska et al., 2022). Begall and Mills (2011) focus on three distinct perceived job characteristics to capture different fertility reactions by employed women: the first, *perceived work control*, is based on the following six features: pace of work, daily organisation of work, power in company policy decisions, requirement to learn new skills, if the job offers variety in tasks and challenges, and if tasks are not closely supervised. The second factor is *job strain* or the feeling that there is never enough time to finish tasks. Finally, *work–family conflict* comprises the following four aspects: worrying about work problems when not working, feeling too tired after work to enjoy things one would like to do at home, finding the job prevents from spending time with a partner or family, and perceiving that the partner or the family get fed up with the pressures of the respondent’s job. They further incorporate a weight based on the importance placed on being able to combine family and work when choosing a job.

Arguably, many emotions associated with employment uncertainty are not directly capturable. Vignoli et al. (2020b) use subjective well-being (SWB) as a proxy for unobservable job characteristics, positing that SWB is negatively affected by unemployment, tenuous employment, and the distinct characteristics of particular jobs that make people feel unstable. They argue that employment uncertainty may be highly age- and career-specific; part-time or fixed-contract work may give younger, less established workers opportunities to be flexible in their careers, temporarily increasing SWB, but that flexibility can turn to insecurity as they age and would like to start a family. The authors use SWB to capture the age when flexible working situations no longer increase SWB but infringe upon it.

### Career making

Employment uncertainty could be the perception of working in a position below career ambitions (e.g., individuals perceive that they are on the wrong career trajectory with their current employment). While this might directly relate to income, it can also concern a position’s prestige and the perception of advancement opportunities. Schmitt (2012) examines educational qualifications and first job characteristics to test if not having a perceived sufficiently prestigious job affects fertility outcomes. He creates an index to see if individuals were underqualified, properly qualified, or overqualified for their first position. Thus, the hypothesis is that establishing a level of employment stability often starts at the relative level of first employment, and individual sense of stability depends on if individuals believe their qualifications match the job prestige. In this case, subjective perception is derived from objective measures and not directly asked.

### Subjective perceptions of security

The most direct way to capture forward-looking subjective perceptions of employment uncertainty is to ask job security questions in surveys. Golsch (2003) includes the perception of job satisfaction^8^ as a way to assess job security. Hanappi et al. (2017) expand on this by explicitly asking about job security^9^ to evaluate the risk of becoming unemployed in the following 12 months. They create an indicator to see if perceived insecurity rose or declined between waves as well as an indicator for changes to or from fixed-term contracts. They add an indicator for change in job security by the respondents’ partners.

Kreyenfeld (2010) incorporates employment status and subjective perception of job security. Respondents are asked if they are “worried”, “somewhat worried”, or “not worried” about losing their job. This is interacted with the respondents’ employment status to see if there is heterogeneity of entry into motherhood among employed individuals based on a perception that their employment may not last. This measure only looks at those in the labour market. Bhaumik and Nugent (2011) utilise two questions to assess perceptions of job security of respondents in and out of the labour market: one for employed persons about the risk of losing their job (very concerned, somewhat concerned, or not concerned at all) and one for unemployed individuals about the ease of finding employment (easy, difficult, or almost impossible). They combine these two items to create a 6-point scale, with *1* being “employed and secure” and *6* being “unemployed with a low perceived chance of finding a job.” The logic is that the middle scores, *3* or *4*,^10^ are where individuals face the most employment uncertainty.

Fahlén and Oláh (2018) combine perceived job security with perceived income security to more comprehensively capture the subjective threat of insecurity through self-evaluation questions about respondents’ employment circumstances and economic resources. Perceived job security is evaluated with the statement “[m]y job is secure”,^11^ while perceived income security is evaluated according to the perception of household financial resources.^12^ Along with income, van Wijk et al. (2021) use two types of subjective forms of job security^13^ to mediate the effect of temporary contracts. Both variables are measured as dummies: *1* if the person perceives job insecurity and *0* if not.

Various studies have used perceptions of financial security as a measure of general economic uncertainty (Kreyenfeld, 2005, 2010, 2016; Testa & Basten, 2014). However, since childbearing/childrearing carries significant financial costs, there is an endogenous relationship between the answers individuals give in the survey and their likelihood of getting pregnant in the next year. Hofmann and Hohmeyer (2013) examine perceived economic uncertainty using survey data.^14^ They then use an instrumental variable approach with survey waves before and after an announced German unemployment benefit reform to remove the endogeneity related to subjective perceptions of financial security and fertility behaviours. They additionally stratify the sample into household types: dual-income, male main breadwinner (women work at least part-time), and specialised male breadwinner (women are non-working) to study how perceived financial stress affects individuals within the financial context that fertility decisions are made. While this method does not directly test for employment uncertainty, the authors relate household type and financial stress directly to employment situations.

### Perception of resilience

Uncertainty can be seen as not only the fear of losing current employment, but also as the fear of not being able to replace it quickly. Gatta et al. (2021) incorporate the perception of job security (likelihood of still having a job in the next 6 months) with an additional theoretical idea of resilience, captured by the perceived ability to find a new job in case of job loss. They control for the subjective differences in perception of resilience with an additional question about the individual’s risk tolerance. Schmitt (2021) uses a measure of risk tolerance^15^ paired with employment status to capture the subjective negative effect of unemployment or suboptimal employment on first birth.

### Experimental methods

To look at how regional and national level attitudes influence individuals’ notion of uncertainty, Guetto et al. (2022) expand the narrative framework into an experimental framework using short-term fertility intentions. They split the sample into five groups. Each group received a different prognosis of future economic situations post-COVID. In a follow-up interview a few months later, each respondent received one of several fabricated news stories about the COVID-19 pandemic, describing various lengths of return to normality following a lockdown. The goal was to examine if consumed media (or communal perceptions of economic/employment uncertainty) affects individual levels of uncertainty about fertility intentions, later repeated in Italy and Norway (Vignoli et al., 2022; Lappegård et al., 2022). This experimental approach is innovative and its larger application for measuring the effect of communal perceptions of uncertainty still needs to be worked out in the field.

### Macro-measures

The most commonly used macro-level measures for employment uncertainty are national and local unemployment rates. Unemployment rates can signify the capacity to find or change employment. Goldstein et al. (2013) use lagged unemployment rates. Adsera (2011a) applies 12-month backdated female unemployment rates as well as the national long-term unemployment rate interacted with the female labour force participation rate. Kravdal (2002) uses the regional unemployment rate to see how regional and sector-specific unemployment affects fertility behaviour, differentiating between agricultural, manufacturing, and service sectors. Pailhé and Solaz (2012) use gender-specific regional unemployment rates as a proxy for the individual perceived risk of unemployment. Fahlén and Oláh (2018) use unemployment rates plus a measure of employment protection legislation (as measured by the OECD) that combines individual and collective termination and temporary employment legislation.^16^ Campisi et al. (2022) use the share of economically inactive people, rather than an unemployment rate, in an attempt to capture those that turn to education or family life when labour market prospects look bad.

The share of workers in part-time work, the public sector, self-employment, or fixed-term contracts varies significantly across high-income countries. These differences may help explain the observed differences in fertility patterns across countries. Adsera (2011a) uses the share of public sector, self-employment, and part-time employment to look at the effect of contractual agreements on birth outcomes across countries. Part-time employment is interacted with female labour force participation rates to evaluate the relative importance of part-time work as a strategy for reconciling work and family.

## Discussion

Beyond measure choice, there are several aspects researchers should keep in mind when considering how to model the relationship between employment uncertainty and fertility. First, this section discusses population heterogeneity and different reactions to employment uncertainty based on sex, age, educational attainment, and parity. Next, it suggests considerations of the contextual factors in which employment uncertainty takes place. Finally, it reflects on modelling choice.

### Population heterogeneity

The literature finds that men have a negative association between unemployment, part-time work, fixed-term contracts, being in unskilled occupations, and fertility. However, through the 1990s, in most contexts, it appeared that for women there existed a *substitution effect,* in which unemployment was positively associated with fertility. The theorised reason is that if women are outside of the labour market they have a lower opportunity cost to have a(nother) child. However, this relationship seems to have reversed across a variety of low-fertility contexts (Alderotti, 2022; Doepke et al., 2022; Schmitt, 2021; Yu & Sun, 2018). This change suggests that the *income effect* has become more important. Women need stable employment, and the income it brings, to have children. They may also be more career-oriented and prioritise the ability to participate in the labour market. The sex-specific employment relationship appears to be disappearing. However, early evidence suggests this may not be consistent across the entire population.

The age at which employment uncertainty occurs appears to influence individuals’ responses to it. It seems that unemployment has a significant relationship with lowering fertility for individuals older than 30 years (Goldstein et al., 2013; Miettinen & Jalovaara, 2020). The age of first birth continues to increase across Europe. Hence, more individuals are making their fertility decisions at later ages. We can therefore logically conclude that individuals who delay fertility decisions to their 30s have a higher risk of having negative fertility outcomes in the case of economic downturns and employment uncertainty. What we do not know is if individuals/couples are delaying fertility because of early career employment precarity or opt for more precarious employment because they already intend to delay family formation. When stratifying by both age and educational attainment further heterogeneity is observed (Miettinen & Jalovaara, 2020). Unemployment has a positive association with becoming a mother for low educated women, although this decreases with age. On the other hand, for high educated women, unemployment is uniformly negative regardless of age. Education also precedes the type of jobs available to an individual, meaning that different educational groups will experience distinct types of employment uncertainty. Finally, the age of leaving education is causally linked to the age of first birth (Black et al., 2008; Skirbekk et al., 2004). Thus, the expansion of education and the associated establishment of a career are already directly interacting with the biological pressure of realising fertility desires.

A key insight from demography and family sociology is that first birth is a special transition into parenthood and the calculations individuals/couples make are different from those for higher order birth. Most studies model these transitions separately with exceptions coming mainly from causal models (Boca et al., 2005; Hofmann & Hohmeyer, 2013; Huttunen & Kellokumpu, 2016). It is also possible to model all births together while still including parity-specific transitions by including parity as a covariate (Kravdal, 2002). Of the articles cited in "Measures" section only six explicitly model higher-order births (Table 1). It appears that employment uncertainty has a stronger negative association with first birth than higher parities. However, our better understanding of the relationship between first birth and employment uncertainty is likely related to the fact that much more attention has been given to the transition to parenthood. Papers modelling first and higher births together could help to see if the effects are consistent across parity. We do not know if the relationship between higher-order birth and women’s employment uncertainty has changed over the past few decades as we have observed for first birth.

### Contextual factors

Social policy plays an intermediating role between employment regimes, macroeconomic conditions, and fertility (Sobotka et al., 2011). Most approaches to examining social policy have used cross-country comparisons. However, the evidence for the influence of social policy on fertility remains inconclusive and insufficient (Gauthier, 2007). Causally linking social policies with fertility has proven to be difficult (Kreyenfeld, 2021). Social policies like child tax benefits, parental leave, and expanded childcare likely influence individuals’ decisions about if their current employment provides enough income and stability to pursue their fertility desires. There is a considerable need to tease out the contextual effects of such measures. How much social policy overcomes global forces, decreasing employment uncertainty, is not clear.

Countries vary in the level of employment protection legislation (EPL) which influences the type of labour market individuals encounter. EPL could work in two directions (Bastianelli et al., 2022). Strong EPL creates an insider–outsider labour market where those on the outside have to accept precarious employment. Outsiders would largely include younger workers and the less educated. It is possible outsiders have lower fertility. On the other hand, strong EPL may reduce employment uncertainty by reducing potential job displacement, the use of fixed-term or temporary contracts, and reducing perceived employment uncertainty, increasing fertility. Early work connecting EPL and fertility finds that reducing EPL negatively affects fertility (De Paola et al., 2021). Although much more exploration is needed on this front. In general, little empirical work has been done to connect individuals to their meso- and macro-level employment regimes.

Studies focusing on macroeconomic trends and micro-level employment are still largely walled off from each other. Several studies show that in low-fertility countries TFR is cyclical with the macroeconomic situation (Comolli et al., 2021; Neels et al., 2013; Örsal & Goldstein, 2018). The TFR tends to decline in economic downturns presumably due to increased unemployment, increased precarious employment, increased financial uncertainty, and declining consumer confidence. It appears that the negative association is stronger for first births than higher-order births (Comolli, 2017; Goldstein et al., 2013). These studies can only hypothesise on micro-level mechanisms at play. More studies are emerging that joint model micro- and macro-indicators examine the interaction between the two levels (Comolli, 2021; de Lange et al., 2014). Many micro-level studies have observation periods overlapping periods of economic downturn. These periods are often not explicitly modelled, with few exceptions (Alderotti et al., 2022; Kristensen & Lappegård, 2022). Importantly, perceived employment uncertainty could be the result of business cycle downturns. Using year/period dummies to control for period effects helps control for macroeconomic influence. Applying matching techniques and separating economic downturns from economic upturns may help parse these effects.

### Model choice

The first decision in modelling is the outcome variable choice. This is often dictated by data availability, with researchers relying more heavily on measures of fertility preferences when vital statistics data are unavailable. Studying fertility preference allows researchers to observe if there are mismatches between desired and realised fertility. This includes studying the ideal number of lifetime children as well as short-term fertility intentions. However, studies linking women’s employment and fertility preferences show little association (Kuhnt et al., 2017, 2021). Additionally, individuals adjust their fertility intentions and ideal family sizes to reflect their partnership and life circumstances. Reverse causality also poses a problem with these measures. Individuals may change their fertility preference to reflect their employment situations or may select their employment situation in concordance with their fertility preferences. This makes establishing causality difficult.

Papers examining the relationship between employment uncertainty and fertility outcomes have largely used event history analysis. These models permit the use of unbalanced panels, overcoming issues of attrition that plague panel surveys. Furthermore, event history analysis allows for the use of time-varying covariates, giving researchers access to richer measures that more fully capture fluctuations in employment uncertainty. Many of the measures discussed in "Measures" section depend on event history analysis to study the dynamic nature of employment. However, modelling employment as a time-varying covariate risks endogenous selection bias, in which past employment affects the confounders of current employment (Elwert & Winship, 2014). Furthermore, these models cannot show causality. Similar to fertility preference, most standard models of fertility outcomes suffer from the existence of reverse causality. One method to minimise this is to attempt to control for all confounders. However, this is restricted by access to variables and has been inconsistently applied in studies (Matysiak & Vignoli, 2008). Finally, event history analysis might capture postponed but not abandoned births. More work is needed to examine the role employment uncertainty plays on completed fertility. An alternative to event history analysis is causal models. However, births are fairly uncommon events making the data demands quite large (for a discussion on the use of causal modelling in fertility research please see Kreyenfeld, 2021). With births being relatively rare events, many of the most common causal models struggle to show significant results. This may change with larger, high-quality datasets coming out in the future.

## Conclusion

Where does employment uncertainty fit in the larger landscape of explanations for the continued decline in TFR in low-fertility countries? As previously discussed, several forms of employment uncertainty are linked to negative fertility outcomes. However, since most studies only test one conceptualisation of employment uncertainty it can be hard to draw valid conclusions about the whole concept. There exists a large amount of empirical evidence that unemployment negatively associates with fertility behaviour. However, does this mean that employment uncertainty, rather than something like the loss of income, is the mechanism behind the association? This remains an open question. What makes employment uncertainty particularly difficult to parse from other mechanisms is that it may also be negatively associated with fertility through other life course domains. For example, men appear to have a negative relationship with fertility and employment uncertainty, due to their attractiveness in the marriage market (Kalmijn, 2011; Oppenheimer, 1988). Individuals who struggle to find stable, well-enumerated employment may not be able to make other important transitions like finding a partner, getting married, and buying a home. The expansion of education plays a role, pushing out the age of the first career-job, and changing the composition of the country. Individuals change their fertility desires in response to their life situation and surrounding context.

Alternative explanations for the decline in TFR include the double burden that many employed women face due to a stalled gender revolution (McDonald, 2013). We have seen that employment is now a prerequisite for motherhood in many low-fertility countries. Being in the labour market while simultaneously doing more to rear children and maintain a household makes reconciling work and family hard. An increase in male participation in domestic labour could change this equation, possibly altering the employment/fertility relationship. Another explanation is that attitudes towards self-fulfilment, family, and career may have changed (Lesthaeghe, 2010). The relative roles of employment, leisure, and family may change life priorities away from a more structured life path. Thus, other desires may interact with both employment and fertility decisions. Finally, we note that only recently has more attention been given to the interlinking role of income and employment uncertainty. Recent studies imply that measures that negatively associate with fertility like part-time work, fixed/temporary contracts, and perceived employment uncertainty may be secondary to the influence of household income (Miettinen & Jalovaara, 2020; van Wijk et al., 2021). If households have stable or sufficient income, women’s employment situation may not matter much for fertility behaviour.

This paper reviews several conceptualisations and measures of employment uncertainty with the goal of helping push the field beyond extrapolating from employment status and contract type. It highlights several open questions within the field. Still, this overview paper is not without its limitations. Employment uncertainty studies tend to be limited to a few high-income countries. Much of the work on employment uncertainty takes a life course perspective, popularised throughout Europe (Huinink & Kohli, 2014). For example, several East Asian countries with sustained low fertility do not have the longitudinal datasets necessary for life course studies. On the other hand, in countries that have more recently undergone the demographic transition, employment may still be a relatively small contributor to overall fertility. Expanding the scope of country contexts is an important next step. Additionally, as more high-quality longitudinal data sources become available the possibilities for measuring and modelling employment uncertainty will increase. Finally, employment uncertainty is highly linked to economic uncertainty. There are several measures available that aim to explain the relationship between economic uncertainty and fertility. We did not include them in this overview to maintain a focused perspective.

## Data Availability

Not applicable.

## Appendix

See Table 1.

**Table 1:** List of sources and measures of employment uncertainty

Paper	Measure	Sex*	Parity/behaviour	Country	Lagged	Obj./Subj	Micro/macro	Method
Adsera (2011a)	*Macro:* Female unemployment rate, national long-term unemployment rate Share of public sector, self-employment, or part-time worker Interacted with female labour force participation rate	F	Second	Multiple	12 months	Obj	Macro	Cox proportional hazard model
Adsera (2011b)	*Employment status:* Working or unemployed/inactive	F	Second Third	Multiple	7 months	Obj	Micro	Cox proportional hazard model
Alderotti (2022)	*Employment status:* Working or unemployed/inactive	F	First	Italy	9 months	Obj	Micro	Cox proportional hazard model
Baizán (2005)	*Employment status:* Employed, unemployed, student, or housewife; public/private sector; permanent, temporary, or self-employed; full/part-time	F	Second +	Denmark Italy Spain UK	9 months	Obj	Micro	Piecewise constant exponential model
Baizán (2009)	*Employment status:* Same variables as in Baizán (2005), but using time-varying variables	F	First Second +	Spain	9 months	Obj	Micro	Piecewise constant exponential model with fixed regional effects
Barbieri et al. (2015)	*Employment status:* 4 employment categories: permanent, self-employed, atypical, and non-standard Binary indicator of *insecure work* (atypical or non-standard) and *secure work* (permanent or self-employed)	F	First	Italy Spain	12 months	Obj	Micro	Discrete time event history analysis
Begall and Mills (2011)	*Job characteristics:* 3 measures: perceived work control, job strain, importance placed on combining work and family	F	Intentions	Multiple	–	Subj	Micro	Multilevel logit regression
Bernardi and Nazio (2005)	*Occupational class:* 8 occupational classifications split into economically secure/insecure	B	First	Italy	9 months	Obj	Micro	Piecewise constant exponential model
Bhaumik and Nugent (2011)	*Subjective perception of security:* Indicator combing subjective measure of risk of losing job (employed) and ease of finding job (unemployed)	F	Likelihood of childbirth	East and West Germany	–	Subj	Micro	Random effect probit regression
Busetta et al. (2019)	*Number and duration of job spells:* Indicator combining number, duration, pairwise proximity, and recentness of years with discontinuous employment	C	Intentions	Italy	–	Obj	Micro	Ordered logit regression
Campisi et al. (2022)	*Macro:* Municipal-level economic inactive rate	–	Total and age-specific fertility rates	Denmark Finland Norway Sweden	–	Obj	Macro	Random effect spatial panel regression
Ciganda (2015)	*Number and duration of job spells:* Indicator combining number and duration of spells in “unstable” states	B	First	France	?	Obj	Micro	Sequence analysis, Logit regression
Comolli (2021)	*Employment status:* 7 category classification of couple-level employment and income situation	C	First	United States	Previous wave (t-1)	Obj	Micro	Linear probability model
Fahlén and Oláh (2018)	*Subjective perception of security:* Job security, income security	B	Intentions	Multiple	–	Subj	Micro	Logit regression
-	*Macro:* Employment Protection Legislation Index	–	–	–	–	Obj	Macro	–
Gatta et al. (2021)	*Perception of resilience:* Likelihood to lose job, likelihood to find a new job in case of job loss	B	Intentions	Italy	–	Subj	Micro	Ordered logit regression
Goldstein et al. (2013)	*Macro:* Unemployment rate	–	Total and Age-specific Fertility Rates	Multiple	12 months	Obj	Macro	Fixed-effects model
Golsch (2003)	*Employment status:* Contract length + full/part-time	B	First	Spain	12 months	Obj	Micro	Logit regression
-	*Subjective perception of security:* Job satisfaction	–	–	–	–	Subj	Micro	–
Guetto et al. (2022)	*Experimental methods:* Participants read news about various economic forecasts	B	Intentions	Italy	–	Subj	Macro	Stepwise OLS regression
Hanappi et al. (2017)	*Subjective perception of security:* Job security Indicator of rising or falling insecurity (for individual and partner)	C	Intentions to outcomes	Switzerland	–	Subj	Micro	Logit regression
Hofmann and Hohmeyer (2013)	*Subjective perception of security:* Financial security	F	Likelihood of becoming pregnant	Germany	12 months	Subj	Micro	Instrumental variable regression
Kravdal (2002)	*Macro:* Regional unemployment rate, sector-specific unemployment rate	B	First Second +	Norway	12 months	Obj	Macro	Poisson continuous time hazard regression
Kreyenfeld (2010)	*Subjective perception of security:* Worry about losing employment	F	First	Germany	9 months	Subj	Micro	Piecewise constant exponential model
Kurowska et al. (2022)	*Job characteristics:* Access to home-based work	B	Intentions	Poland	–	Obj	Micro	Multinomial logit regression
Lappegård et al. (2022)	*Experimental methods:* Participants read news about various economic forecasts	C	Intentions	Norway	–	Subj	Macro	Linear regression
Miettinen and Jalovaara (2020)	*Employment status:* Employment status, unemployed split between short- and long-term	B	First	Finland	7 months	Obj	Micro	Piecewise constant exponential model
Özcan et al. (2010)	*Number and duration of job spells:* Number of months, number of unemployment spells, number of prior job shifts	B	First	Germany	12 months	Obj	Micro	Piecewise constant exponential model
Pailhé and Solaz (2012)	*Employment status:* Long-term, short-term, unemployment, or homemaker Employment status at union formation	B	First completed	France	Previous year (t-1) Fixed	Obj	Micro	Cox proportional hazard model
-	*Number and duration of job spells:* Ratio of years spent in unemployed/short-term employment over permanent employment since union formation	–	–	–	?	Obj	Micro	–
-	*Macro*: Gender-specific unemployment rates	–	–	–	Previous year (t-1)	Obj	Macro	–
Schmitt (2008)	*Income:* Relative income within partnership Net personal income	B	First	Finland France Germany UK	10 months	Obj	Micro	Piecewise constant exponential model
Schmitt (2008)	*Number and duration of job spells:* Number of long-term employment spells (4 + months)	–	–	–	–	Obj	Micro	Piecewise constant exponential model
Schmitt (2012)	*Job characteristics:* Hours of overtime worked	B	First	Germany UK	10 months	Obj	Micro	Piecewise constant exponential model
-	*Career making:* Index of being underqualified, properly qualified, or overqualified for first position	–	–	–	–	Obj.^1^	Micro	–
Schmitt (2021)	*Perception of resilience:* A question of risk tolerance is paired with employment status	B	First	Germany	10 months	Subj	Micro	Cox proportional hazard model
-	*Number and duration of job spells:* Indicator of months unemployed over total number of months	–	–	–	–	Obj	Micro	–
van Wijk et al. (2021)	*Subjective perception of security:* Individual perceives risk of losing job and worries about losing job	F	First	Netherlands	9 months	Obj	Micro	Discrete time event history analysis
Vignoli et al. (2020b)	*Job characteristics:* Subjective well-being	C	Intentions (Childless, Higher parity)	Multiple	–	Subj	Micro	Logit regression
Vignoli et al. (2020c)	*Employment status:* Employment contact type	B	First	Italy	–	Obj	Micro	Average treatment effect for the treated
Wood and Neels (2017)	*Employment status:* 11 categories combining partnership status and partner’s employment status 5 categories of women's employment status	F	First Second Third	Belgium	12 months	Obj	Micro	Discrete time event history analysis

* F = females, B = both sexes, C = couples.1 The index is derived from objective measures with an explicit goal of capturing unreportedsubjective perceptions

## Footnotes

1 Screening criteria include: low-fertility context, use of quantitative method, and outcome variable fertility behaviour or intention. Eligibility criteria focuses on if the principle explanatory variable relates to examining if uncertain employment negatively associates with fertility behaviour/intention.

2 Self-employment includes business owners with or without dependent employees, freelance workers, and entrepreneurs. For some individuals, self-employment gives flexibility and work control that is difficult to achieve with dependent employment. Self-employed individuals might also be able to take advantage of increased economic opportunities. However, self-employment may also be a reaction to a poor labour market. There is also a growing trend by industries to re-classify dependent workers as independent contractors to get around paying taxes and benefits. Employment uncertainty as a result of self-employment is largely dependent on social welfare and employment regulation. It is also likely gender specific, with self-employment increasing the income-opportunity trade-off.

3 Atypical employment is defined as a fixed-term contract or pseudo-self-employed—performing a job with a self-employed contract in a subordinate position. Non-standard jobs are mainly seasonal or “off-the-books”.

4 International Standard Classification of Occupation (ISCO) by the International Labour Organization. Barbieri et al. (2015) use the ISCO-08 framework.

5 Women’s partnership + employment status was measured in 2001. They then use discrete time hazard models to estimate timing of first, second, and third births for 2002–2005.

6 He distinguishes between the following states: *student*, *military service*, *full-time employment*, *self-employed*, *part-time employment*, *on leave*, *helping at home*, *unemployed*, *retired*, *inactive*, *sick*, or *other.* For men, he defines unemployment and part-time employment as unstable states, while only unemployment is classified as unstable for women. This is because a majority of men reported that being in part-time employment was involuntary, while women may use part-time employment as a way to reconcile career and family duties. Although, there is evidence that women make up a larger share on involuntary part-time workers due to care responsibilities and occupational sector (Pech et al., 2021).

7 In a hypothetical scenario where two respondents spent the same amount of time unemployed but the first respondent had 10 spells, while the second respondent only had two spells, the first respondent would have an indicator that signifies a higher level of instability.

8 From the Spanish European Community Household Panel (ECHP): “How satisfied are you with your present job or business in terms of earnings, hours of work, working conditions, etc.”.

9 “Would you say that your job is very secure, quite secure, a bit insecure or very insecure?”.

10 *3* is coded as individuals who are employed but “very concerned” about losing their job, while *4* is coded as individuals who are unemployed who can “easily” find employment.

11 On a 5-point Likert scale.

12 “How do you feel about your household’s income nowadays?” The possible answers are: living comfortably, coping, finding it difficult, or finding it very difficult.

13 If individuals perceive that they are at risk of losing their job (*cognitive job insecurity*) and if they worry about losing their job (*affective job insecurity*).

14 From the German Socioeconomic Panel “What is your attitude towards your own economic situation?” with the options of: very concerned, somewhat concerned, or not concerned at all.

15 “How do you see yourself: Are you generally a person who is fully prepared to take risks or do you try to avoid taking risks?” Using an 11-point Likert scale from 0 (“unwilling to take risks”) to (“completely willing to take risk”).

16 The OECD uses 24 measures of the strictness of employment protection for both individuals and collective dismissals to create an indicator of the level of legal employment protection in each country. Specific details can be found here: https://www.oecd.org/els/emp/oecdindicatorsofemploymentprotection.htm.

## References

[CR1] Adsera A (2004). Changing fertility rates in developed countries. The impact of labor market institutions. Journal of Population Economics.

[CR2] Adsera A (2011). Where are the babies? Labor market conditions and fertility in Europe. European Journal of Population/revue Européenne De Démographie.

[CR3] Adsera A (2011). The Interplay of Employment Uncertainty and Education in explaining Second Births in Europe. Demographic Research.

[CR4] Alderotti G (2022). Female employment and first childbirth in Italy: What news?. Genus.

[CR5] Alderotti G, Mussino E, Comolli CL (2022). Natives’ and migrants’ employment uncertainty and childbearing during the great recession: A comparison between Italy and Sweden. European Societies.

[CR6] Alderotti G, Vignoli D, Baccini M, Matysiak A (2021). Employment Instability and Fertility in Europe: A Meta-Analysis. Demography.

[CR7] Anderson CJ, Pontusson J (2007). Workers, worries and welfare states: Social protection and job insecurity in 15 OECD countries. European Journal of Political Research.

[CR8] Baizán, P. (2005). *The impact of labour market status on second and higher-order births. A comparative study of Denmark, Italy, Spain and United Kingdom*. http://repositori.upf.edu/handle/10230/267

[CR9] Baizán P (2009). Regional child care availability and fertility decisions in Spain. Demographic Research.

[CR10] Barbieri P, Bozzon R, Scherer S, Grotti R, Lugo M (2015). The rise of a Latin model? Family and fertility consequences of employment instability in Italy and Spain. European Societies.

[CR11] Bastianelli, E., Guetto, R., & Vignoli, D. (2022). The impact of labour market deregulation reforms on fertility in Europe. *Econometrics Working Papers Archive*, Art. 2022_04. https://ideas.repec.org//p/fir/econom/wp2022_04.html

[CR12] Beck U (1992). Risk society: Towards a New Modernity.

[CR13] Becker GS (1960). An Economic Analysis of Fertility, Demographic and economic change in developed countries: A conference of the Universities. National Bureau Commitee for Economic Research.

[CR14] Beckert J (2016). Imagined futures: Fictional expectations and capitalist dynamics.

[CR15] Begall K, Mills M (2011). The impact of subjective work control, job strain and work–family conflict on fertility intentions: A European comparison. European Journal of Population/revue Européenne De Démographie.

[CR16] Bernardi F, Nazio T (2005). Globalization and the transition to adulthood in Italy. Globalization, Uncertainty and Youth in Society: the Losers in a Globalizing World.

[CR17] Bernardi L, Huinink J, Settersten RA (2019). The life course cube: A tool for studying lives. Advances in Life Course Research.

[CR18] Bernardi L, Mynarska M, Rossier C (2015). Uncertain, changing and situated fertility intentions. Reproductive decision-making in a macro-micro perspective.

[CR19] Bhaumik SK, Nugent JB (2011). Real options and demographic decisions: Empirical evidence from East and West Germany. Applied Economics.

[CR20] Black SE, Devereux PJ, Salvanes KG (2008). Staying in the Classroom and Out of the Maternity Ward? The Effect of Compulsory Schooling Laws on Teenage Births. The Economic Journal.

[CR21] Blossfeld H-P, Klijzing E, Mills M, Kurz K (2006). Globalization, uncertainty and youth in society: The losers in a globalizing world.

[CR22] Boca DD, Pasqua S, Pronzato C (2005). Fertility and Employment in Italy, France, and the UK. Labour.

[CR23] Brehm U, Schneider NF (2019). Towards a Comprehensive Understanding of Fertility: The Model of Dyadic Pathways. Comparative Population Studies.

[CR24] Busetta A, Mendola D, Vignoli D (2019). Persistent joblessness and fertility intentions. Demographic Research.

[CR25] Campisi N, Kulu H, Mikolai J, Klüsener S, Myrskylä M (2022). A spatial perspective on the unexpected nordic fertility decline: the relevance of economic and social contexts. Applied Spatial Analysis and Policy.

[CR26] Cheng GH-L, Chan DK-S (2008). Who Suffers More from Job Insecurity? A Meta-Analytic Review. Applied Psychology.

[CR27] Ciganda D (2015). Unstable work histories and fertility in France: An adaptation of sequence complexity measures to employment trajectories. Demographic Research.

[CR28] Comolli CL (2017). The fertility response to the Great Recession in Europe and the United States: Structural economic conditions and perceived economic uncertainty. Demographic Research.

[CR29] Comolli CL (2021). Couples’ paid work, state-level unemployment, and first births in the United States. Demographic Research.

[CR30] Comolli CL, Neyer G, Andersson G, Dommermuth L, Fallesen P, Jalovaara M, Jónsson AK, Kolk M, Lappegård T (2021). Beyond the Economic Gaze: Childbearing During and After Recessions in the Nordic Countries. European Journal of Population.

[CR31] Davidson P (1996). Reality and economic theory. Journal of Post Keynesian Economics.

[CR32] de Lange M, Wolbers MHJ, Gesthuizen M, Ultee WC (2014). The Impact of Macro- and Micro-Economic Uncertainty on Family Formation in The Netherlands. European Journal of Population.

[CR33] De Paola M, Nisticò R, Scoppa V (2021). Employment Protection and Fertility Decisions: The Unintended Consequences of the Italian Jobs Act. Economic Policy.

[CR34] Doepke M, Hannusch A, Kindermann F, Tertilt M (2022). The economics of fertility: A new era.

[CR35] Elster J (2009). Excessive ambitions. Capitalism and Society.

[CR36] Elwert F, Winship C (2014). Endogenous selection bias: the problem of conditioning on a collider variable. Annual Review of Sociology.

[CR37] Esser I, Olsen KM (2012). Perceived job quality: autonomy and job security within a multi-level framework. European Sociological Review.

[CR38] Fahlén S (2013). Capabilities and Childbearing Intentions in Europe: The association between work–family reconciliation policies, economic uncertainties and women’s fertility plans. European Societies.

[CR39] Fahlén S, Oláh LS (2018). Economic uncertainty and first-birth intentions in Europe. Demographic Research.

[CR40] Gatta A, Mattioli F, Mencarini L, Vignoli D (2021). Employment uncertainty and fertility intentions: Stability or resilience?. Population Studies.

[CR41] Gauthier AH (2007). The impact of family policies on fertility in industrialized countries: A review of the literature. Population Research and Policy Review.

[CR42] Gebel M, Giesecke J (2011). Labor Market Flexibility and Inequality: The Changing Skill-Based Temporary Employment and Unemployment Risks in Europe. Social Forces.

[CR43] Goldstein JR, Kreyenfeld M, Jasilioniene A, Örsal DK (2013). Fertility reactions to the “Great Recession” in Europe: Recent evidence from order-specific data. Demographic Research.

[CR44] Golsch K (2003). Employment flexibility in Spain and its impact on transitions to adulthood. Work, Employment and Society.

[CR45] Guetto R, Bazzani G, Vignoli D (2022). Narratives of the future and fertility decision-making in uncertain times. An application to the COVID-19 pandemic. Vienna Yearbook of Population Research.

[CR46] Hacker JS (2019). The great risk shift: The new economic insecurity and the decline of the American dream.

[CR47] Hanappi D, Ryser V-A, Bernardi L, Le Goff J-M (2017). Changes in employment uncertainty and the fertility intention–realization link: An analysis based on the Swiss household panel. European Journal of Population.

[CR48] Hellstrand J, Nisén J, Miranda V, Fallesen P, Dommermuth L, Myrskylä M (2021). Not just later, but fewer: Novel trends in cohort fertility in the Nordic countries. Demography.

[CR49] Hofmann B, Hohmeyer K (2013). Perceived economic uncertainty and fertility: Evidence from a labor market reform. Journal of Marriage and Family.

[CR50] Huinink J, Kohli M (2014). A life-course approach to fertility. Demographic Research.

[CR51] Huttunen K, Kellokumpu J (2016). The effect of job displacement on couples’ fertility decisions. Journal of Labor Economics.

[CR52] Iceland J (2005). Measuring poverty: theoretical and empirical considerations. Measurement: Interdisciplinary Research and Perspectives.

[CR53] Jahedi S, Méndez F (2014). On the advantages and disadvantages of subjective measures. Journal of Economic Behavior & Organization.

[CR54] Kalmijn M (2011). The influence of men’s income and employment on marriage and cohabitation: testing oppenheimer’s theory in Europe. European Journal of Population / Revue Européenne De Démographie.

[CR55] Kohler H-P, Billari FC, Ortega JA (2002). The emergence of lowest-low fertility in Europe during the 1990s. Population and Development Review.

[CR56] Kravdal Ø (2002). The impact of individual and aggregate unemployment on fertility in Norway. Demographic Research.

[CR57] Kreyenfeld, M. (2005). *Economic uncertainty and fertility postponement—Evidence from German panel data. MPIDR Working Paper 2005*.

[CR58] Kreyenfeld M (2010). Uncertainties in female employment careers and the postponement of parenthood in Germany. European Sociological Review.

[CR59] Kreyenfeld M (2016). Economic uncertainty and fertility. Social Demography Forschung an der Schnittstelle von Soziologie und Demografie.

[CR60] Kreyenfeld M (2021). Causal modelling in fertility research: a review of the literature and an application to a parental leave policy reform. Comparative Population Studies.

[CR61] Kreyenfeld M, Andersson G, Pailhé A (2012). Economic uncertainty and family dynamics in Europe: Introduction. Demographic Research.

[CR62] Kristensen AP, Lappegård T (2022). Unemployment and fertility: The relationship between individual and aggregated unemployment and fertility during 1994–2014 in Norway. Demographic Research.

[CR63] Kuhnt, A.-K., Kreyenfeld, M., & Trappe, H. (2017). Fertility ideals of women and men across the life course. In *Childlessness in Europe: Contexts, Causes, and Consequences* (pp. 235–251). Springer, Cham

[CR64] Kuhnt A-K, Minkus L, Buhr P (2021). Uncertainty in fertility intentions from a life course perspective: Which life course markers matter?. Journal of Family Research.

[CR65] Kurowska, A., Matysiak, A., & Osiewalska, B. (2022). Working from home during Covid-19 pandemic and changes to fertility intentions among parents. In *Working Papers* (No. 2022–22; Working Papers). Faculty of Economic Sciences, University of Warsaw. https://ideas.repec.org/p/war/wpaper/2022-22.html 10.1007/s10680-023-09678-z PMC10581933 37847441

[CR66] Lappegård T, Kristensen AP, Dommermuth L, Minello A, Vignoli D (2022). The impact of narratives of the future on fertility intentions in Norway. Journal of Marriage and Family.

[CR67] Lesthaeghe R (2010). The Unfolding Story of the Second Demographic Transition. Population and Development Review.

[CR68] Matysiak A, Sobotka T, Vignoli D (2020). The Great Recession and fertility in Europe: A sub-national analysis. European Journal of Population.

[CR69] Matysiak A, Vignoli D (2008). Fertility and women’s employment: A meta-analysis. European Journal of Population/revue Européenne De Démographie.

[CR70] Mayer KU (2009). New Directions in Life Course Research. Annual Review of Sociology.

[CR71] McDonald P (2013). Societal foundations for explaining low fertility: Gender equity. Demographic Research.

[CR72] Miettinen A, Jalovaara M (2020). Unemployment delays first birth but not for all Life stage and educational differences in the effects of employment uncertainty on first births. Advances in Life Course Research.

[CR73] Mills M, Blossfeld H-P (2003). Globalization, uncertainty and changes in early life courses. Zeitschrift Für Erziehungswissenschaft.

[CR74] Neels K, Theunynck Z, Wood J (2013). Economic recession and first births in Europe: Recession-induced postponement and recuperation of fertility in 14 European countries between 1970 and 2005. International Journal of Public Health.

[CR75] Oppenheimer VK (1988). A theory of marriage timing. American Journal of Sociology.

[CR76] Örsal DDK, Goldstein JR (2018). The changing relationship between unemployment and total fertility. Population Studies.

[CR77] Özcan B, Mayer KU, Luedicke J (2010). The impact of unemployment on the transition to parenthood. Demographic Research.

[CR78] Pailhé A, Solaz A (2012). The influence of employment uncertainty on childbearing in France: A tempo or quantum effect?. Demographic Research.

[CR79] Pech C, Klainot-Hess E, Norris D (2021). Part-time by Gender, Not Choice: The Gender Gap in Involuntary Part-time Work. Sociological Perspectives.

[CR80] Peters J (2008). Labour market deregulation and the decline of labour power in North America and Western Europe. Policy and Society.

[CR81] Richiardi, M. G., & He, Z. (2019). *Measuring economic insecurity: A review of the literature*. mimeo, ISER, University of Essex.

[CR82] Schmitt C (2012). Labour market integration, occupational uncertainties, and fertility choices in Germany and the UK. Demographic Research.

[CR83] Schmitt C (2021). The impact of economic uncertainty, precarious employment, and risk attitudes on the transition to parenthood. Advances in Life Course Research.

[CR84] Skirbekk V, Kohler H-P, Prskawetz A (2004). Birth month, school graduation, and the timing of births and marriages. Demography.

[CR85] Sobotka T, Skirbekk V, Philipov D (2011). Economic recession and fertility in the developed world. Population and Development Review.

[CR86] Stiglitz, J., Sen, A., & Fitoussi, J.-P. (2009). The measurement of economic performance and social progress revisited. *Reflections and Overview. Commission on the Measurement of Economic Performance and Social Progress, Paris*.

[CR87] Sverke M, Hellgren J, Näswall K (2002). No security: A meta-analysis and review of job insecurity and its consequences. Journal of Occupational Health Psychology.

[CR88] Testa MR, Basten S (2014). Certainty of meeting fertility intentions declines in Europe during the ‘Great Recession’. Demographic Research.

[CR89] van Wijk DC, de Valk HAG, Liefbroer AC (2021). Temporary employment and family formation: An income or insecurity effect?. European Sociological Review.

[CR90] Vignoli D, Bazzani G, Guetto R, Minello A, Pirani E, Schoen R (2020). Uncertainty and Narratives of the Future: A Theoretical Framework for Contemporary Fertility. Analyzing Contemporary Fertility.

[CR91] Vignoli D, Mencarini L, Alderotti G (2020). Is the Effect of Job Uncertainty on Fertility Intentions Channeled by Subjective Well-Being?. Advances in Life Course Research.

[CR92] Vignoli D, Tocchioni V, Mattei A (2020). The impact of job uncertainty on first-birth postponement. Advances in Life Course Research.

[CR93] Vignoli D, Minello A, Bazzani G, Matera C, Rapallini C (2022). Narratives of the future affect fertility: evidence from a laboratory experiment. European Journal of Population.

[CR94] Wood J, Neels K (2017). First a job, then a child? Subgroup variation in women’s employment-fertility link. Advances in Life Course Research.

[CR95] Yu W, Sun S (2018). Fertility responses to individual and contextual unemployment: Differences by socioeconomic background. Demographic Research.

